# The challenges of *in situ* detection for micro- and nanoplastics

**DOI:** 10.1016/j.eehl.2025.100194

**Published:** 2025-10-25

**Authors:** Ye Li, Junjie Zhang, Chu Peng, Li Xu

**Affiliations:** a MOE Key Laboratory of Pollution Processes and Environmental Criteria, College of Environmental Science and Engineering, Nankai University, Tianjin 300071, China; b Department of Plant and Environmental Sciences, University of Copenhagen, 1871 Frederiksberg C, Denmark; c Institute of Quality Standard and Testing Technology, Beijing Academy of Agriculture and Forestry Sciences, Beijing 100097, China

Micro- and nanoplastics (MNPs) are pervasive, mobile pollutants with high bioavailability [1], making *in situ* analysis within organisms crucial to accurately quantify their concentration and map their distribution, as demonstrated by techniques like hyperspectral imaging [2].

However, this goal is hampered by significant biological and technical challenges. Upon entering a biological system, MNPs undergo two key processes that complicate detection. MNPs rapidly adsorb a layer of biomolecules, forming a protein corona that alters their size, charge, and surface chemistry [[3], [4], [5]]. MNPs can be degraded in vivo through enzymatic or oxidative processes, which add new functional groups and fragment them into even smaller, harder-to-detect NPs [6]. These transformations present major obstacles for spectroscopic techniques like μRaman or μFTIR. The protein corona masks intrinsic spectral fingerprints by introducing new peaks and increasing background noise, while biotransformation chemically alters the polymer's original spectral signature. Although emerging label-free techniques such as Optical Photothermal Infrared (O-PTIR) spectroscopy [7] and Atomic Force Microscopy-Infrared (AFM-IR) spectroscopy [8] offer improved spatial resolution, they remain fundamentally susceptible to these same spectral interferences.

In light of these limitations, mass spectrometry imaging (MSI) has emerged as a promising alternative for *in situ* MNPs analysis due to its high sensitivity and molecular specificity [9]. However, MSI techniques also face limitations: the biological matrix can suppress ionization in time-of-flight secondary ion mass spectrometry (TOF-SIMS), desorption electrospray ionization (DESI) is challenged by the hydrophobicity of polymers, and matrix-assisted laser desorption/ionization (MALDI) has sensitivity limitations. Furthermore, techniques like SIMS and MALDI can cause laser degradation and entail high maintenance costs [2,10].

Currently, no single technique is perfect for the *in situ* analysis of MNPs in biological samples. Two strategic pathways could be focused on to mitigate biological interferences. Developing sophisticated data processing methods to carefully isolate the MNPs signal from the biological background. Innovating technologies that can penetrate the surface protein corona or new functional groups to analyze the core polymer material directly, thereby revealing the particle's true chemical identity.

## CRediT authorship contribution statement

**Ye Li:** Investigation, Writing – original draft. **Junjie Zhang:** Conceptualization, Supervision, Writing – original draft, Writing – review & editing. **Chu Peng:** Funding acquisition, Writing – review & editing. **Li Xu:** Conceptualization, Funding acquisition, Supervision, Writing – review & editing.

## Declaration of competing interests

The authors have declared no conflicts of interest.
